# Oleate Hydratase (OhyA) Is a Virulence Determinant in Staphylococcus aureus

**DOI:** 10.1128/Spectrum.01546-21

**Published:** 2021-11-24

**Authors:** Christopher D. Radka, Justin L. Batte, Matthew W. Frank, Jason W. Rosch, Charles O. Rock

**Affiliations:** a Department of Infectious Diseases, St. Jude Children’s Research Hospital, Memphis, Tennessee, USA; Emory University School of Medicine

**Keywords:** *Staphylococcus aureus*, oleate hydratase, hydroxy fatty acids, unsaturated fatty acids, virulence, soft tissue infection, virulence determinants

## Abstract

Staphylococcus aureus is an important pathogen that relies on a variety of mechanisms to evade and counteract the immune system. We show that S. aureus uses oleate hydratase (OhyA) to convert host *cis*-9 unsaturated fatty acids to their 10-hydroxy derivatives in human serum and at the infection site in a mouse neutropenic thigh model. Wild-type and Δ*ohyA* strains were equally infective in the neutropenic thigh model, but recovery of the Δ*ohyA* strain was 2 orders of magnitude lower in the immunocompetent skin infection model. Despite the lower bacterial abundance at the infection site, the levels of interleukin 6 (IL-6), monocyte chemoattractant protein 1 (MCP-1), IL-1β, and tumor necrosis factor alpha (TNF-α) elicited by the Δ*ohyA* strain were as robust as those of either the wild-type or the complemented strain, indicating that the immune system was more highly activated by the Δ*ohyA* strain. Thus, OhyA functions to promote S. aureus virulence.

**IMPORTANCE** The oleate hydratase protein family was discovered in commensal bacteria that utilize host unsaturated fatty acids as the substrates to produce a spectrum of hydroxylated products. These hydroxy fatty acids are thought to act as signaling molecules that suppress the inflammatory response to create a more tolerant environment for the microbiome. S. aureus is a significant human pathogen, and defining the mechanisms used to evade the immune response is critical to understanding pathogenesis. S. aureus expresses an OhyA that produces at least three 10-hydroxy fatty acids from host unsaturated fatty acids at the infection site, and an S. aureus strain lacking the *ohyA* gene has compromised virulence in an immunocompetent infection model. These data suggest that OhyA plays a role in immune modulation in S. aureus pathogenesis similar to that in commensal bacteria.

## OBSERVATION

Commensal organisms of the gut microbiome contain a family of flavin adenine dinucleotide-dependent oleate hydratase genes (*ohyA*) that produce a spectrum of hydroxylated fatty acids (*h*FA) from host unsaturated fatty acids (1). Evidence is accumulating that OhyA-derived *h*FA function to suppress cytokine production and inflammation to create a more tolerant environment for the commensal bacteria (2–5). Staphylococcus aureus is an important pathogen that deploys an array of virulence factors that engage host immune defenses to promote pathogenesis (6–8). S. aureus expresses an OhyA that catalyzes water addition to *cis*-9 double bonds (9) and protects against palmitoleic acid (16:1) (10), an antimicrobial fatty acid produced by the innate immune system (11–13). The goal of this study was to determine if OhyA has a role in S. aureus pathogenesis and supports the production of *h*FA at the infection site.

We found that S. aureus OhyA prefers 18:1 over 18:2 as the substrate based on the described *in vitro* assay (10). Pure OhyA assayed using [^14^C]oleate (18:1) or [^14^C]linoleate (18:2) as the substrate yielded specific activities of 8.25 ± 1.45 and 1.19 ± 0.05 nmol/min/mg, respectively. *h*FA production by S. aureus grown with equal amounts of 18:1 and 18:2 produced 10-hydroxyoctadecanoic acid (*h*18:0) and 10-hydroxy-*cis*-12-octadecenoic acid (*h*18:1) in the 7:1 ratio expected from the enzymology (Fig. S1A). *h*FA were measured by liquid chromatography-tandem mass spectrometry (LC-MS/MS) as their 3-picolylamide derivatives using an *m/z* of 109.0 generated from the loss of the common picolylamide moiety (14). The picolylamide approach circumvents the misrepresentation of *h*FA abundance based on measurements using unique ions generated from breakage at the hydroxyl group position (15). The high efficiency of detecting the *m/z* of 297.1/185.1 Q1/Q3 ion pair from *h*18:1 compared to that of detecting the *m/z* of 271.1/185.1 and *m/z* of 299.1/185.1 Q1/Q3 ion pairs arising from *h*16:0 and *h*18:0, respectively, gives a false view of the relative abundance of the two *h*FA (Fig. S1B). The conclusion that *h*18:1 is the most abundant *h*FA produced by the gut microbiome is based on the latter technique (15).

**FIG 1:**
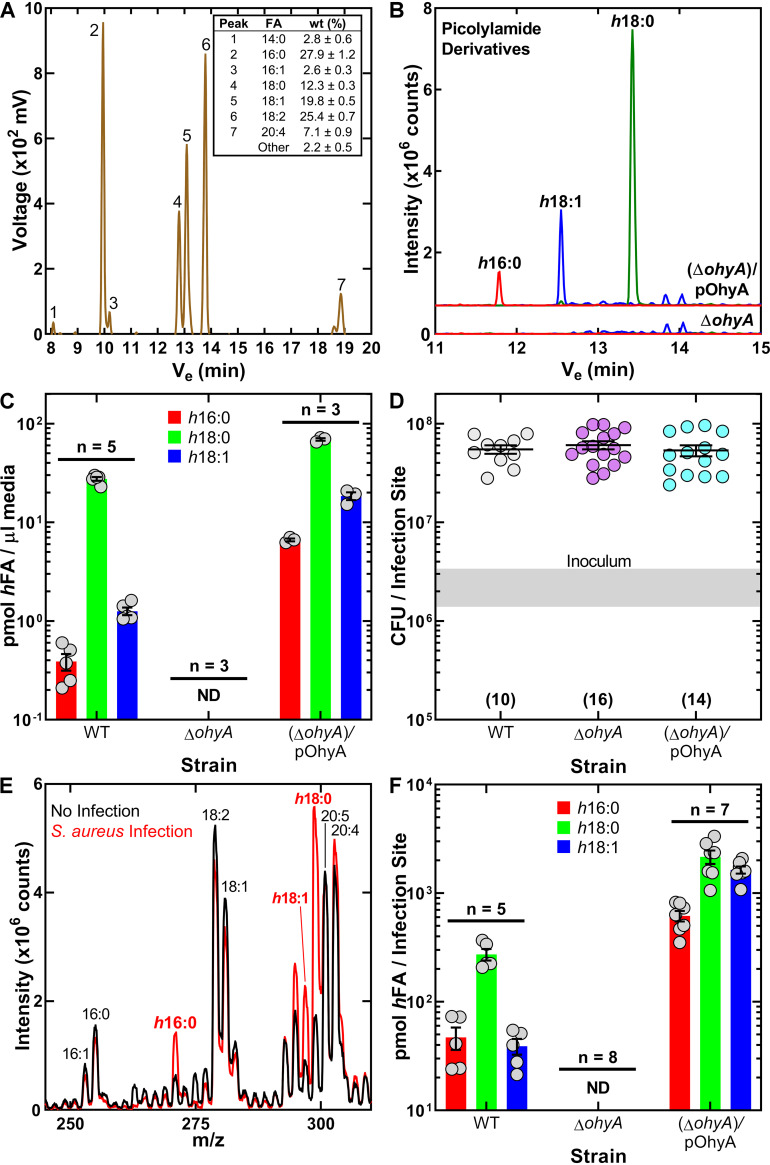
OhyA-dependent *h*FA formation in human serum and the neutropenic thigh infection model. (A) A representative gas chromatogram illustrating the fatty acid composition of the human serum lot with the average fatty acid composition from three replicates (inset). (B) Representative LC-MS/MS chromatograms of picolylamide derivatized *h*FA recovered from the medium following growth of S. aureus strains PDJ68 (Δ*ohyA*) and PDJ68 (Δ*ohyA*)/pOhyA in 50% human serum. (C) Quantification of *h*FA recovered from the medium following growth of S. aureus strains AH1263 (WT), PDJ68 (Δ*ohyA*), and PDJ68 (Δ*ohyA*)/pOhyA in 50% human serum. (D) Enumeration of the bacteria recovered from infected neutropenic thighs. The gray shaded bar represents the range of initial inoculum. Numbers of animals are in parentheses. (E) Representative total ion chromatograms of the fatty acid fraction in the LC-MS/MS analysis of mock-infected (black) and infected (red) neutropenic thighs. (F) Quantification of *h*FA recovered from neutropenic thighs infected with the strain set. ND means <5 pmol. Mean ± standard error of the mean (SEM).

Wild-type S. aureus strain AH1263 and its derivatives, PDJ68 (Δ*ohyA*) and PDJ68 (Δ*ohyA*)/pOhyA (10), were grown in 50% human serum to stationary phase to assess the capability of S. aureus to produce *h*FA in the presence of a mixture of mammalian lipids. The total fatty acid composition of the serum lot shows that the OhyA substrates 18:1 and 18:2 were present in equal amounts, whereas palmitoleate (16:1) was an order of magnitude less abundant (Fig. 1A). However, most of these FA are esterified and not available to OhyA unless first released by S. aureus lipases. Geh is a major extracellular lipase that is known to release FA from serum triacylglycerols for incorporation into S. aureus phospholipids (16). An isogenic strain lacking Geh produces significantly less *h*FA in human serum than either the wild-type or complemented strains (Fig. S2). Geh is only one of many lipases and phospholipases that could contribute to OhyA substrate availability in environments where their specific lipid substrates are abundant. A representative LC-MS/MS analysis of *h*FA produced by strain PDJ68 (Δ*ohyA*)/pOhyA grown in 50% serum illustrates the raw ion current from the *h*FA region of the gradient between 11 and 15 min that was absent in experiments with strain PDJ68 (Δ*ohyA*) (Fig. 1B). Experiments using internal standards showed that *h*18:0 was the major *h*FA produced, reaching a concentration of 27.56 ± 1.21 μM (Fig. 1C). *h*18:1 was the next most abundant, and *h*16:0 was the least abundant. *h*FA production by strain PDJ68 (Δ*ohyA*) was not detected (Fig. 1C). OhyA expression in strain PDJ68 (Δ*ohyA*)/pOhyA resulted in an increase in the levels of all *h*FA. These data show the OhyA-dependent production of *h*FA when grown in human serum.

A neutropenic thigh infection model was used to address the formation of *h*FA *in vivo*. This model was selected to assess *h*FA formation *in vivo* in the presence of equal numbers of bacteria at the infection site. There was no significant difference in the bacterial titers among the strains 24 h after infection (Fig. 1D). We first used shotgun lipidomics profiling (17) to determine if there are changes to the composition of the free fatty acid fraction in the thigh following infection. A comparison of the mock-infected to infected thigh samples showed the appearance of three new peaks that correspond in molecular weight to *h*16:0, *h*18:0, and *h*18:1 (Fig. 1E). Quantitation of the *h*FA composition using picolylamide derivatization showed that the wild-type strain produced predominantly *h*18:0, 7-fold less *h*18:1, and 6-fold less *h*16:0 (Fig. 1F). *h*FA were not detected in thighs inoculated with the Δ*ohyA* strain. *h*FA abundance in thighs infected with the Δ*ohyA*/pOhyA strain was higher than that in thighs infected with the wild type. These data show that *h*FA formation at the infection site is OhyA-dependent.

**FIG 2:**
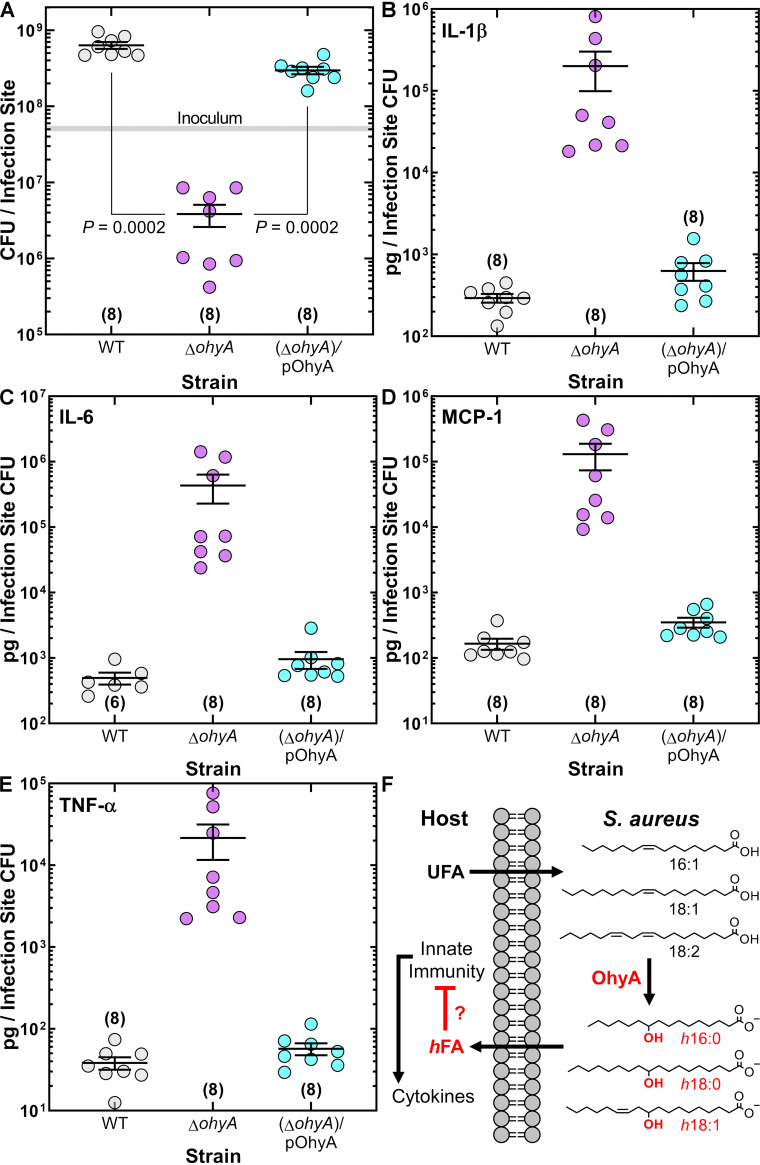
OhyA is a virulence determinant in an SSTI infection model. (A) Enumeration of the bacteria recovered from the infection site. S. aureus strains AH1263 (WT), PDJ68 (Δ*ohyA*), and PDJ68 (Δ*ohyA*)/pOhyA were used to infect mice by subcutaneous infection. Mock-infected mice were given an injection of sterile phosphate-buffered saline (PBS). Kruskal-Wallis test determined whether overall differences between groups have statistical significance, and *P* values were calculated using Mann-Whitney test. The gray shaded bar represents the range of initial inoculum determined by serial dilution. (B to E) Measurements of cytokine analytes that were recovered from the infection sites in the SSTI model. Data are normalized to the number of bacteria recovered. The cytokine levels per infection site are shown in Figure S3. (F) Model for OhyA-dependent *h*FA production at the infection site. Unsaturated *cis*-9 fatty acids (UFA) (16:1, 18:1, 18:2) are converted to hydroxy fatty acids (*h*FA) (*h*16:0, *h*18:0, *h*18:1) by OhyA. *h*FA are released into the extracellular environment. This process inactivates the antimicrobial fatty acids (16:1 and 18:2) and generates mediators that inhibit cytokine production by mechanisms that remain to be delineated. Numbers of animals are in parentheses.

The impact of *ohyA* deletion on virulence was assessed in an immunocompetent skin/soft tissue (SSTI) infection model that showed that the wild-type and complemented strains established an infection but the bacterial burden from Δ*ohyA* knockout was 2 orders of magnitude lower (Fig. 2A). The impact of OhyA expression on the formation of selected cytokines was assessed using a mouse-specific Milliplex cytokine assay platform to measure levels of proinflammatory cytokines that are produced in response to infection (18–20). Although all three strains elicited large, comparable elevations in cytokines (Fig. S3), the immune response to the Δ*ohyA* strain was 2 orders of magnitude higher than that to the others when the data were normalized to the number of cells present (Fig. 2B to E). These data suggest that OhyA suppresses cytokine production.

**Conclusions.** This work establishes OhyA as a determinant of virulence in S. aureus. The importance of OhyA to S. aureus pathogenesis is corroborated by Malachowa et al. (21), who identified a gene they called *sok* that is required for virulence in a rabbit endocarditis infection model. At the time, *sok* was a gene of unknown function, but we now know that it corresponds to *ohyA*. A model for the OhyA-dependent metabolism of host unsaturated fatty acids is diagrammed in Figure 2F. Host unsaturated fatty acids are hydroxylated by OhyA. The *h*FA are not utilized by the pathogen; rather, they are released into the environment. Purified OhyA is a soluble protein, but imaging experiments suggest that it is membrane associated *in vivo* (21) and OhyA is enriched in S. aureus exosomes (22), showing that OhyA is also exported from the cells where it can act on host unsaturated fatty acids. The challenge ahead is to define the mechanism(s) by which the *h*FA interact with the immune system. The inactivation of antimicrobial fatty acids is one clearly identified mechanism (10), but the specifics of how *h*FA interfere with TLR signaling and other arms of the innate immune response remain to be elucidated. PPARγ activation has an established role in regulating host lipid metabolism and inflammation (23), and *h*FA are known agonists of this transcriptional regulator (24). GPR40 and GRP120 are engaged by *h*18:1 (3, 25), but it is not obvious how these nutrient sensors (26) would affect S. aureus virulence. Most intriguing are the published reports of *h*FA suppression of cytokine formation in response to lipopolysaccharide (LPS) and Toll-like receptor (TLR) activation (2–5). More work is needed to define the step(s) in the TLR signaling pathway modulated by *h*FA.
