# Supplementary material for: Oleate Hydratase (OhyA) Is a Virulence Determinant in Staphylococcus aureus
Source: Microbiol Spectr. 2021 Nov 24;9(3):e01546-21. doi: 10.1128/Spectrum.01546-21 (PMC8612147; doi:10.1128/Spectrum.01546-21)
Supplement: SUPPLEMENTAL FILE 1 — Supplemental material. Download SPECTRUM01546-21_Supp_1_seq4.pdf, PDF file, 0.4 MB [file spectrum01546-21_supp_1_seq4.pdf]

Oleate hydratase (OhyA) is a virulence determinant in *Staphylococcus aureus*

Christopher D. Radka, Justin L. Batte*, Matthew W. Frank, Jason W. Rosch,
and Charles O. Rock#

Supplementary Materials

Methods

Materials. All chemicals and reagents were reagent grade or better. Discovery DSC-NH2 solid phase extraction columns were from Millipore Sigma and Acquity UPLC BEH HILIC, 1.7 μm, 2.1 x 150 mm column was from Waters. Solvents were HPLC-grade and obtained from Millipore-Sigma or Fisher unless otherwise indicated.

Bacterial strains. The strains used in this study were strain AH1263, *S. aureus* (wild-type) (1); strain PDJ68 (Δ*ohyA*), an *ohyA* knockout derivative of strain AH1263 (2); and strain PDJ68 (Δ*ohyA*)/pOhyA, the Δ*ohyA* knockout strain harboring an *ohyA* expression vector (2). Strain PDJ171 (Δ*geh*) was prepared starting with strain AH1263 and the *geh* gene removed by allelic replacement as described (3). The removal of the gene was confirmed by PCR using primers SaGeh-Ex-F (5′-gtggcttctcgtttgttg) and SaGeh-Ex-R(5′-ctattgtctacatccacggtg) that yields a 4070 bp band in wild-type and a 2068 bp product in the Δ*geh* strain. The *geh* gene was cloned into pPJ480, a *S. aureus* expression plasmid that drives gene expression from the *sarA* promoter (2) to create pGeh.

Neutropenic thigh infection model. The neutropenic thigh model is an approach that enables reproducible replication of *S. aureus* following inoculation to evaluate pathogenesis in *S. aureus* (4-9). The cyclophosphamide regimen consistently induced profound neutropenia (<10 neutrophils/mm^3). Groups of 8-week-old BALB/c female mice (Jackson Laboratory) per group were treated with a cyclophosphamide regimen optimized to consistently induce neutropenia (<10 neutrophils/mm^3) (9). Cyclophosphamide (150 mg/kg) was intraperitoneal injected 5 days prior

to infection followed by another cyclophosphamide (100 mg/kg) injection 2 days prior to infection. Bacteria were grown in Luria broth overnight at 37°C and back diluted in fresh medium and allowed to grow to an OD_{600} = 0.4. Bacteria were washed twice with sterile phosphate buffered saline (PBS) and suspended into fresh PBS to obtain an inoculum of ~2 x 10^6 CFU/50 µl. Bacteria were introduced via intra-muscular injection of 50 µl wild-type *S. aureus* (AH1263) or its respective mutants in PBS. Mock infections were carried out by injecting 50 µl of sterile PBS. Bacteria were enumerated to determine the exact number of bacteria in the infection dose. At 24 h post infection, thigh muscles were removed and homogenized in 1 ml ice-cold PBS. Homogenate samples were serially diluted and plated on mannitol salt agar selection plates to enumerate bacterial burden in the host tissue.

Skin-soft tissue (SSTI) infection model. The mouse SSTI model was developed to investigate factors related to staphylococcal pathogenesis of skin and soft tissue infections, including the ability to adapt to environmental changes and the production of specific bacterial molecules that contribute to virulence (10). Bacteria were enumerated to determine the exact number of bacteria in each infection dose. The SSTI model used 8-week-old female Balb/C mice that were injected subcutaneously with 5 x 10^7 CFUs and the infection allowed to proceed for 24 hours. Mock infections were carried out by injecting sterile PBS. The infected tissues were excised and homogenized in 1 ml of ice-cold PBS for cytokine analysis and determination of the bacterial burden by serial dilution and plating.

Lipid extractions. Two individual thighs from neutropenic mice were pooled together, homogenized, and the lipids from 100 µl of the homogenate supernatant were extracted using the Bligh and Dyer method (11). The lipid extracts were dried under N_2, resuspended and analyzed by LC-MS/MS as described (12). The ion current in the free fatty acid peak was measured and reported in Fig. 1E. To quantify the *h*FA, the tissue homogenates were spiked with [U-^{13}C]18:1 as the internal standard, and the lipids were extracted (11). The fatty acid fraction was isolated

using a solid phase column (Discovery DSC-NH2, 500 mg) conditioned with 8 ml of hexane (12-14). Non-polar lipids were eluted with 6 ml of 2:1 (v/v) chloroform:isopropanol; fatty acids were eluted with 6 ml of ether + 2% acetic acid; PC and PE were eluted with 6 ml of methanol; and PG and PI were eluted with 6 ml of chloroform:methanol:0.8 M sodium acetate 60:30:4.5 (v/v/v). The fatty acid fraction was dried under N_2 and the free fatty acids were converted to their 3-picolylamide derivatives (15) and analyzed by LC-MS/MS as described below. Alternately, the *h*FA were analyzed without derivatization by LC-MS/MS in the negative mode (16) as described below.

Lipid mass spectrometry. Lipid extracts were resuspended in chloroform/methanol (1:1). FA and *h*FA were analyzed using a Shimadzu Prominence UFLC attached to a QTrap 4500 equipped with a Turbo V ion source (Sciex). Samples (5 μl) were injected onto an Acquity UPLC BEH HILIC, 1.7 μm, 2.1 x 150 mm column (Waters) at 45°C with a flow rate of 0.2 ml/min. Solvent A was acetonitrile, and solvent B was 15 mM ammonium formate, pH 3. The HPLC program was the following: starting solvent mixture of 96% A/4% B; 0 to 2 min, isocratic with 4% B; 2 to 20 min, linear gradient to 80% B; 20 to 23 min, isocratic with 80% B; 23 to 25 min, linear gradient to 4% B; 25 to 30 min, isocratic with 4% B. The QTrap 4500 was operated in the Q1 negative mode. The ion source parameters for Q1 were as follows: ion spray voltage, -4,500 V; curtain gas, 25 psi; temperature, 350°C; ion source gas 1, 40 psi; ion source gas 2, 60 psi; and declustering potential, -40 V. The system was controlled and analyzed by the Analyst software (Sciex).

Picolylamide-*h*FA were analyzed using a Shimadzu Prominence UFLC attached to a QTrap 4500 equipped with a Turbo V ion source (Sciex). Samples (5 μl) were injected onto an XSelect HSS C18, 2.5 μm, 3.0 x 150 mm column (Waters) at 45°C with a flow rate of 0.4 ml/min. Solvent A was 0.1% formic acid in water, and solvent B was acetonitrile with 0.1% formic acid. The HPLC program was the following: starting solvent mixture of 70% A/30% B; 0 to 5 min, isocratic with 30% B; 5 to 15 min, linear gradient to 100% B; 15 to 23 min, isocratic with 100% B; 23 to 25 min,

linear gradient to 30% B; 25 to 30 min, isocratic with 30% B. The QTrap 4500 was operated in the positive mode, and the ion source parameters for the picolylamide-*h*FA multiple reaction monitoring (MRM) parameters were: ion spray voltage, 5,500 V; curtain gas, 15 psi; temperature, 300°C; ion source gas 1, 15 psi; ion source gas 2, 20 psi; declustering potential, 25 V, and a collison energy, 40 V. MRM masses (Q1/Q3) were: picolylamide-*h*16:0, 363.1/109.0; picolylamide-*h*18:0, 391.1/109.0, picolylamide-*h*18:1, 389.1/109.0, and picolylamide-[U-^{13}C]18:1, 391.1/109.0. The system was controlled by the Analyst software (Sciex) and analyzed with MultiQuant™ 3.0.2 software (Sciex). The amount of each *h*FA was calculated based on the known amount of [U-^{13}C]18:1 spiked into the sample at the beginning of the sample preparation (Cambridge Isotopes Labs). The reliable detection limit was 5 pmoles/infection site and 0.01 pmoles/μl in the in vitro experiments.

*h*FA was analyzed using a Shimadzu Prominence UFLC attached to a QTrap 4500 equipped with a Turbo V ion source (Sciex). Samples (5 μl) were injected onto an XSelect HSS C18, 2.5 μm, 3.0 x 150 mm column (Waters) at 45°C with a flow rate of 0.4 ml/min. Solvent A was water, and solvent B was acetonitrile. The HPLC program was the following: starting solvent mixture of 60% A/60% B; 0 to 1 min, isocratic with 40% B; 1 to 16 min, linear gradient to 100% B; 16 to 25 min, isocratic with 100% B; 25 to 27 min, linear gradient to 40% B; 27 to 31 min, isocratic with 40% B. The QTrap 4500 was operated in the negative mode, and the ion source parameters for the *h*FA MRM masses (Q1/Q3) were: ion spray voltage, -4,500 V; curtain gas, 30 psi; temperature, 320°C; ion source gas 1, 20 psi; ion source gas 2, 35 psi; declustering potential, -25 V, and a collison energy, -35 V. MRM masses (Q1/Q3) were: *h*16:0, 271.1/185.1; *h*18:0, 299.1/185.1, and *h*18:1, 297.1/185.1. The system was controlled by the Analyst software (Sciex) and analyzed with MultiQuant™ 3.0.2 software (Sciex).

OhyA enzyme assays. *S. aureus* OhyA was purified as previously described (17). Enzyme assays using 0, 0.75, 1.5, 3, 6, or 12 μg OhyA/assay were carried out in 20 μl reactions containing

150 mM NaCl, 0.2 mg/ml fatty acid-free bovine serum albumin, 50 mM FAD, 10 mM DTT, 50 mM potassium phosphate buffer, pH 6.0, and 20 μM [1-^{14}C]18:1(9*Z*) (specific activity 59 mCi/mmol) or 20 μM [1-^{14}C]18:2(9*Z*, 12*Z*) (specific activity 54.5 mCi/mmol). Reactions incubated at 37°C for 10 min and then were spotted on Silica Gel H TLC plates that were developed in chloroform:methanol (90:10, v/v). The [^{14}C]hydroxy fatty acids were detected using a Typhoon PhosphorImager and quantified using ImageQuant. OhyA specific activities with 18:1 and 18:2 were calculated from the linear region of the protein curves.

Equimolar 18:1/18:2 competition cell assay. Wild-type *S. aureus* was back diluted to OD_{600}=0.05 in 1% tryptone broth containing 1% dimethyl sulfoxide from an overnight culture, and grown to OD_{600}=0.5 at 37°C. An equimolar mixture of 18:1 and 18:2 was added to a final concentration of 20 μM and the cells were grown for an additional 2 h. Cells were pelleted and the lipids were extracted from 1 ml of the culture supernatant using the Bligh and Dyer method (11). Samples were spiked with [U-^{13}C]18:1 as the internal standard. The lipid extract was dried under N_2, resuspended in 800 μl $CHCl_3$:MeOH (1:1), and the total mixture was loaded on a Discovery® DSC-NH2 SPE cartridge (Supelco). *h*FA were isolated by solid phase chromatography, dried under N_2, and either directly analyzed by LC-MS/MS as their free acids in the negative mode or converted to their picolylamide derivatives and analyzed by LC-MS/MS in the positive mode as described above.

Growth in human serum. Wild-type *S. aureus* or the respective mutants were normalized to OD_{600}=0.5 in 1:1 1% tryptone broth:50% human serum (MilliporeSigma cat no. H4522, lot no. SLCD1948) from an overnight culture grown in 1% tryptone broth. Cells were grown for an additional 8 hrs. Cells were pelleted, the free fatty acids were isolated from the cell supernatant by solid phase extraction and derivatized with an internal standard for quantitation as stated above. Hydroxy fatty acids were quantitated using LC-MS/MS. Methyl esters were made from lipid extracts of human serum and separated for identification and quantitation using a Hewlett-

Packard model 5809A gas chromatograph equipped with a flame ionization detector and 30 m × 0.53 mm × 0.50 mm Agilent J&W DB-225 GC Column as described (18).

Quantitation of cytokine protein levels in tissue samples. Cytokine protein levels were analyzed using the MILLIPLEX® Multiplex Assay, a bead-based fluorescence immunoassay method (MilliporeSigma) (19, 20). The infected tissues were excised and homogenized in 1 ml of ice-cold PBS for cytokine analysis. Four analytes (IL-1β, IL-6, MCP-1, and TNF-α) were measured in 25 μl aliquots of the tissue homogenates. All reagents were used before their stated expiration date and all assay steps were performed according to the manufacturer instructions. Data are presented as either absolute amounts per infection site or normalized to the number of bacteria recovered at the infection site.

Statistical analyses. All data are given as mean values ± standard error of the mean (S.E.M.) from at least three independent experiments. Statistical analyses were performed using GraphPad Prism v.9.1.1 software. Non-parametric ANOVA (Kruskal-Wallis) testing was used to determine if a significant difference was present among groups. If a significant difference was detected, then non-parametric ad hoc Mann-Whitney tests were used for follow-up with statistical comparisons between groups and the calculation of *P*-values.

Ethics statement. All work with *S. aureus* described herein were done in accordance with protocols approved by the Institutional Biosafety Committees at St. Jude Children's Research Hospital. All animal experiments were performed with prior review and approval by the St. Jude Institutional Animal Care and Use Committee. All mice were maintained in biosafety level 2 (BSL2) facilities in accordance with IACUC protocol number 566-100442-10/16.

AUTHOR CONTRIBUTIONS

C.D.R., J.L.B., and C.O.R. conceptualization; C.D.R. and J.L.B. formal analysis; C.D.R., J.L.B., M.W.F., J.W.R., and C.O.R. investigation; C.D.R., J.L.B., M.W.F., J.W.R., and C.O.R.

methodology; C.D.R. writing-original draft; C.D.R., J.L.B., M.W.F., J.W.R., and C.O.R. writing-reviewing and editing; C.O.R. resources; C.O.R. supervision; C.D.R. visualization; C.O.R. funding acquisition; C.O.R. project administration.

SUPPLEMENTAL REFERENCES

1. Parsons JB, Broussard TC, Bose JL, Rosch JW, Jackson P, Subramanian C, Rock CO. 2014. Identification of a two-component fatty acid kinase responsible for host fatty acid incorporation by *Staphylococcus aureus*. Proc Natl Acad Sci U S A 111:10532-10537. https://doi.org/10.1073/pnas.1408797111
2. Subramanian C, Frank MW, Batte JL, Whaley SG, Rock CO. 2019. Oleate hydratase from *Staphylococcus aureus* protects against palmitoleic acid, the major antimicrobial fatty acid produced by mammalian skin. J Biol Chem 294:9285-9294. https://doi.org/10.1074/jbc.RA119.008439
3. Bae T, Schneewind O. 2006. Allelic replacement in *Staphylococcus aureus* with inducible counter-selection. Plasmid 55:58-63. https://doi.org/S0147-619X(05)00053-3
4. Lepak AJ, Zhao M, Andes DR. 2017. Comparative pharmacodynamics of telavancin and vancomycin in the neutropenic murine thigh and lung infection models against *Staphylococcus aureus*. Antimicrob Agents Chemother 61:e00281-00217. https://doi.org/10.1128/AAC.00281-17
5. Bulik CC, Okusanya OO, Lakota EA, Forrest A, Bhavnani SM, Hoover JL, Andes DR, Ambrose PG. 2017. Pharmacokinetic-pharmacodynamic evaluation of gepotidacin against Gram-positive organisms using data from murine infection models. Antimicrob Agents Chemother 61:e00115-00116. https://doi.org/10.1128/AAC.00115-16
6. Janardhanan J, Meisel JE, Ding D, Schroeder VA, Wolter WR, Mobashery S, Chang M. 2016. In vitro and in vivo synergy of the oxadiazole class of antibacterials with b-lactams. Antimicrob Agents Chemother 60:5581-5588. https://doi.org/10.1128/AAC.00787-16
7. Lepak AJ, Seiler P, Surivet JP, Ritz D, Kohl C, Andes DR. 2016. In vivo pharmacodynamic target investigation of two bacterial topoisomerase inhibitors, ACT-387042 and ACT-292706, in the neutropenic murine thigh model against *Streptococcus pneumoniae* and *Staphylococcus aureus*. Antimicrob Agents Chemother 60:3626-3632. https://doi.org/10.1128/AAC.00363-16
8. Vogelman B, Gudmundsson S, Turnidge J, Leggett J, Craig WA. 1988. In vivo postantibiotic effect in a thigh infection in neutropenic mice. J Infect Dis 157:287-298.
9. Zuluaga AF, Salazar BE, Rodriguez CA, Zapata AX, Agudelo M, Vesga O. 2006. Neutropenia induced in outbred mice by a simplified low-dose cyclophosphamide regimen: characterization and applicability to diverse experimental models of infectious diseases. BMC Infect Dis 6:55. https://doi.org/10.1186/1471-2334-6-55
10. Malachowa N, Kobayashi SD, Lovaglio J, DeLeo FR. 2019. Mouse model of *Staphylococcus aureus* skin infection. Methods Mol Biol 1960:139-147. https://doi.org/10.1007/978-1-4939-9167-9_12
11. Bligh EG, Dyer WJ. 1959. A rapid method of total lipid extraction and purification. Can J Biochem Physiol 37:911-917.
12. Frank MW, Yao J, Batte JL, Gullett JM, Subramanian C, Rosch JW, Rock CO. 2020. Host fatty acid utilization by *Staphylococcus aureus* at the infection site. mBio 11:e00920. https://doi.org/10.1128/mBio.00920-20

13. Brugger B, Erben G, Sandhoff R, Wieland FT, Lehmann WD. 1997. Quantitative analysis of biological membrane lipids at the low picomole level by nano-electrospray ionization tandem mass spectrometry. Proc Natl Acad Sci U S A 94:2339-2344.
14. Alvarez JG, Touchstone JC. 1992. Separation of acidic and neutral lipids by aminopropyl-bonded silica gel column chromatography. J Chromatogr A 577:142-145.
15. Li X, Franke AA. 2011. Improved LC-MS method for the determination of fatty acids in red blood cells by LC-orbitrap MS. Anal Chem 83:3192-3198. https://doi.org/10.1021/ac103093w
16. Kishino S, Takeuchi M, Park SB, Hirata A, Kitamura N, Kunisawa J, Kiyono H, Iwamoto R, Isobe Y, Arita M, Arai H, Ueda K, Shima J, Takahashi S, Yokozeki K, Shimizu S, Ogawa J. 2013. Polyunsaturated fatty acid saturation by gut lactic acid bacteria affecting host lipid composition. Proc Natl Acad Sci U S A 110:17808-17813. https://doi.org/10.1073/pnas.1312937110
17. Radka CD, Batte JL, Frank MW, Young BM, Rock CO. 2021. Structure and mechanism of *Staphylococcus aureus* oleate hydratase (OhyA). J Biol Chem 296:e100252. https://doi.org/10.1074/jbc.RA120.016818
18. Radka CD, Frank MW, Rock CO, Yao J. 2020. Fatty acid activation and utilization by *Alistipes finegoldii*, a representative Bacteroidetes resident of the human gut microbiome. Mol Microbiol 113:807-825. https://doi.org/10.1111/mmi.14445
19. Guo Q, Zhang J, Li J, Zou L, Zhang J, Xie Z, Fu X, Jiang S, Chen G, Jia Q, Li F, Wan Y, Wu Y. 2013. Forced miR-146a expression causes autoimmune lymphoproliferative syndrome in mice via downregulation of Fas in germinal center B cells. Blood 121:4875-4883. https://doi.org/10.1182/blood-2012-08-452425
20. Pusic K, Aguilar Z, McLoughlin J, Kobuch S, Xu H, Tsang M, Wang A, Hui G. 2013. Iron oxide nanoparticles as a clinically acceptable delivery platform for a recombinant blood-stage human malaria vaccine. FASEB J 27:1153-1166. https://doi.org/10.1096/fj.12-218362

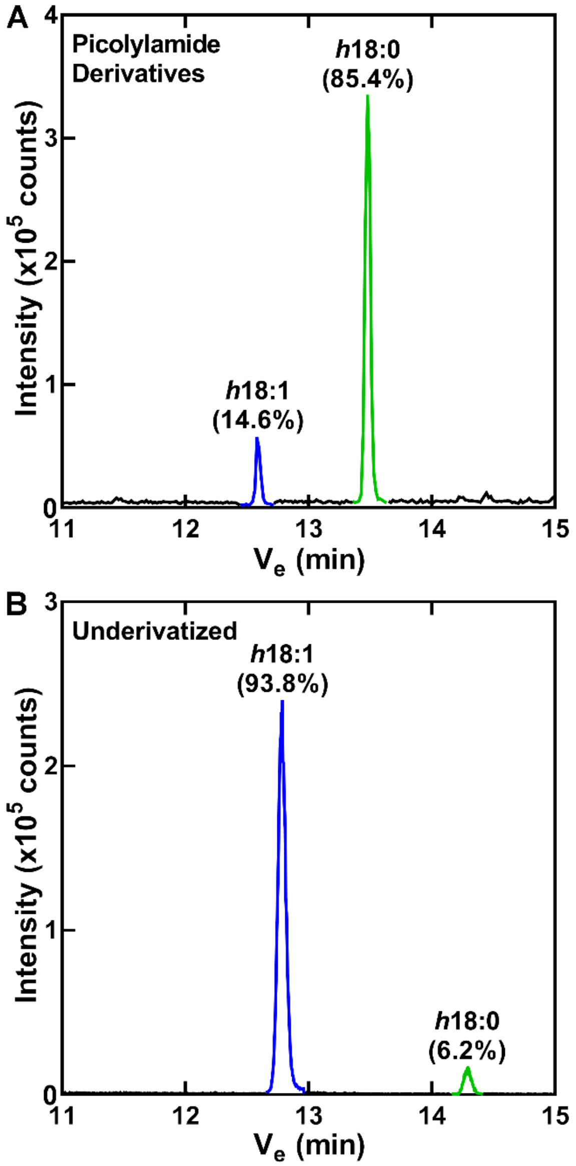

FIG S1 Picolylamine derivatization normalizes hydroxy fatty acid response in mass spectrometry. Fatty acids were extracted from the culture supernatant of *S. aureus* grown with an equimolar mixture of 18:1 and 18:2. The lipids were extracted and the fatty acid fraction isolated by solid phase extraction. (A) Fatty acids were analyzed as their picolylamide derivatives by LC-MS/MS in the positive mode using MRM transitions for *h*18:0 (*m/z* = 391.1/109.0) and *h*18:1 (*m/z* = 389.1/109.0) corresponding to the common loss of *m/z* = 109.0 from the picolylamide moiety (15). (B) Underivatized fatty acids were analyzed by LC-MS/MS in the negative mode using MRM transitions arising from unique ions generated from breakage at the hydroxyl group position to measure *h*18:0 (*m/z* = 299.1/185.1) and *h*18:1 (*m/z* = 297.1/185.1) (16).

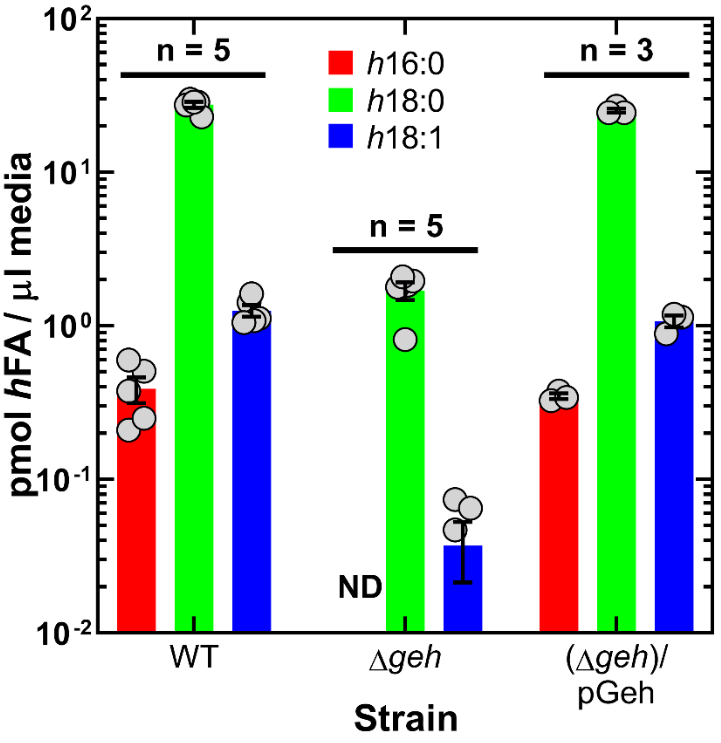

FIG S2 The impact of Geh lipase on *h*FA formation in human serum. Quantification of *h*FA recovered from the media following growth of *S. aureus* strains PDJ171 (Δ*geh*) and PDJ171(Δ*geh*)/pGeh in 50% human serum as compared to AH1263 (WT) (Fig. 1C). ND means < 0.01 pmoles/µl.

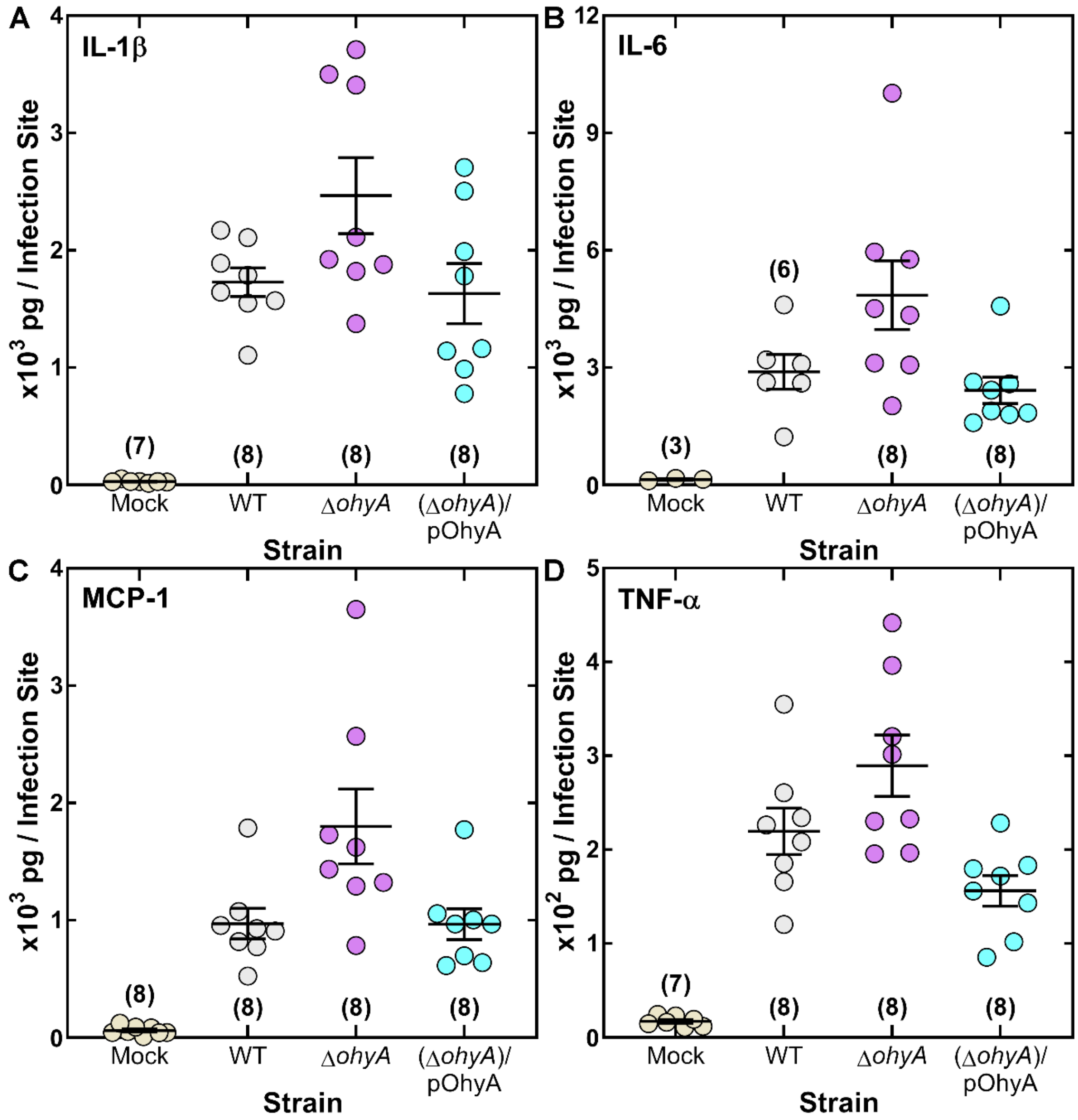

FIG S3 Measurements of cytokine analytes recovered from the SSTI infection site. Cytokines were quantified using the mouse specific cytokine assay system. Data are the total cytokine levels detected from the infection site. (A) IL-1β. (B) IL-6. (C) MCP-1. (D) TNF-α.
